# Supplementary material for: CEBPA-Regulated Expression of SOCS1 Suppresses Milk Protein Synthesis through mTOR and JAK2-STAT5 Signaling Pathways in Buffalo Mammary Epithelial Cells
Source: Foods. 2023 Feb 6;12(4):708. doi: 10.3390/foods12040708 (PMC9955710; doi:10.3390/foods12040708)
Supplement: Supplementary file 1 [file foods-12-00708-s001.zip › Supplementary figures.pdf]

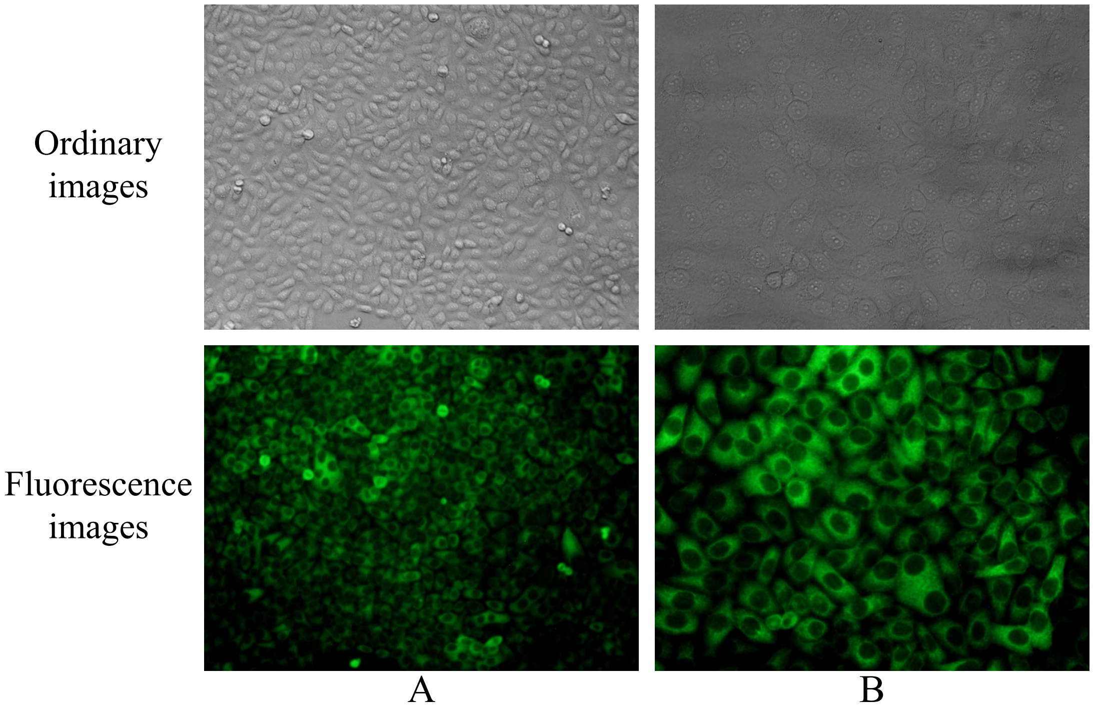

Figure S1. Images of buffalo mammary epithelial cells incubated with anti-cytokeratin 18 monoclonal antibody (A×200, B×400).

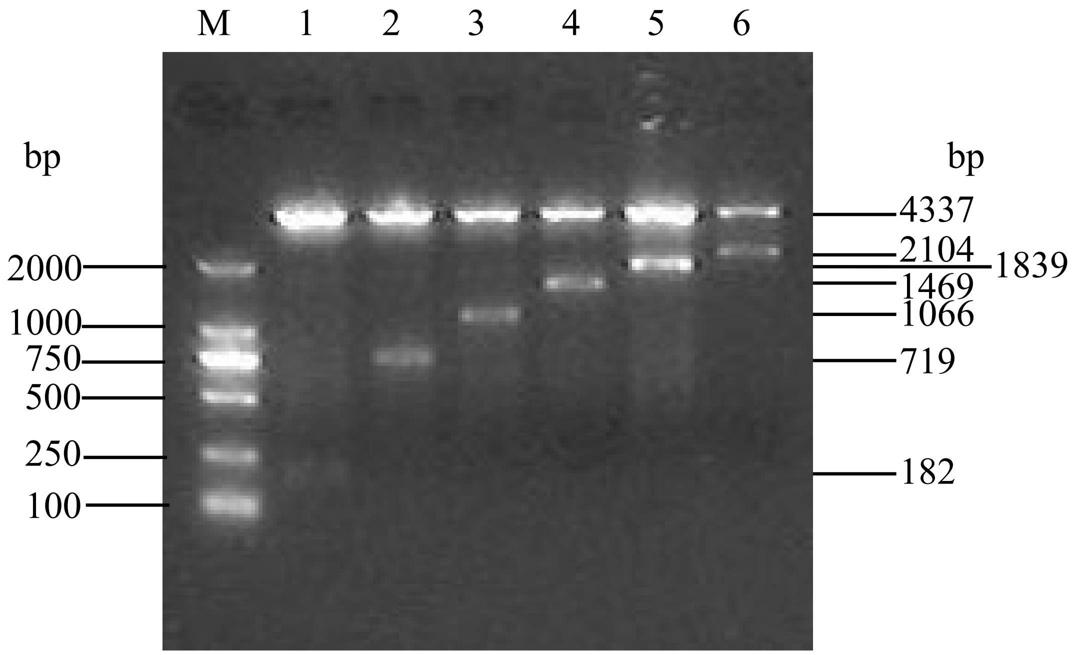

Figure S2. Double-enzyme digestion of 5’ progressive deletion of pGL-SOCS1 recombinant plasmid. M, DL2000 Marker; 1-6, the fragments of *SOCS1* promoter: −77~+105 bp, −614~+105 bp, −961~+105 bp, −1364~+105 bp, −1734~+105 bp and −1999~+105 bp.
